# Nonstereoselective dissipation of sulfoxaflor in different Puer tea processing

**DOI:** 10.1002/fsn3.1789

**Published:** 2020-07-26

**Authors:** Hongcheng Liu, MingMing Jiang, Qiwan Li

**Affiliations:** ^1^ Institute of Quality Standard and Testing Technology Yunnan Academy of Agricultural Science, Supervision and Testing Center for Farm Product Quality Ministry of Agriculture Kunming China; ^2^ School of Pharmaceutical Science & Yunnan Key Laboratory of Pharmacology for Natural Products Kunming Medical University Kunming China

**Keywords:** LC‐MS/MS, Puer tea processing, stereoselective dissipation, sulfoxaflor

## Abstract

The stereoisomer behavior of sulfoxaflor was investigated by liquid chromatography‐tandem mass spectrometry (LC‐MS/MS) during raw Puer tea, ripen Puer tea, and sun‐dry Puer tea processing. The persistence of sulfoxaflor stereoisomers was consistently prolonged with different Puer tea processing from sun‐dry Puer tea, ripen Puer tea to raw Puer tea with *t*_1/2_ 4.0–4.2 hr (sun‐dry Puer tea) to 6.21–7.04 months (raw Puer tea). Three fermentation temperature in ripen Puer tea shown that the sulfoxaflor residue was easy to degrade under low fermentation temperature (37°C). It implied that enzyme catalysis may play an important role in degradation of sulfoxaflor. The no‐enantioselective dissipation of sulfoxaflor was found in different Puer tea processing.

## Graphical abstract

It is first time to determine the stereoselective dissipation of sulfoxaflor enantiomers in sun‐dry Puer tea, raw Puer tea, and ripen Puer tea processing. The persistence of sulfoxaflor stereoisomers was consistently prolonged from sun‐dry Puer tea processing, raw Puer tea to ripen Puer tea with *t*_1/2_ 4.0–4.2 hr to 6.21–7.04 months.

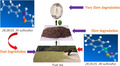

## 1 INTRODUCTION

Puer tea is famous with long history in Yunnan Province, China. Puer tea is classified as a postfermented tea according to the processing technology (Zhang, Zhang, Zhou, Ling, & Wan, 2013). Two main processing are including raw Puer tea and ripen Puer tea (Chinese National standard GB/T 22111‐2008). The raw Puer tea is fermented by nature condition, its history is from long‐distance transport with consign for horse. The ripen Puer tea is from pile‐fermentation with high temperature (<50°C) and high humidity in modern technology. Because the pile‐fermentation processing is more rapid fermented than nature fermentation, so the pile‐fermentation processing is called as ripen Puer tea, the other is called as raw Puer tea. The ripened Puer tea contains lots of microbe will produce flavor compounds during the postfermentation process (Zhang, Li, Ma, & Tu, 2011). Although many studies about the chemistry and biological activities of Puer tea during fermentation are available in the literature (Long et al., 2019), the effects of chiral pesticide degradation in Puer tea processing are still unclear.

Sulfoxaflor, [methyl(oxo)1‐[6‐(trifluoromethyl)‐3‐pyridyl] ethy‐6‐sulfanylidene]cyanamide, is apparently effective for insecticides acting on nAChRs (Cutler et al., 2013; Liu et al., 2010; Xu et al., 2012), and used as insecticides to control *Empoasca pirsuga* Matumura in tea (Chen et al., 2014a). New research has found that some pesticides containing chiral centers will undergo stereo degradation and present potential toxicities (de Albuquerque, Carrão, Habenschus, & de Oliveira, 2018). In some cases, only one of the enantiomers is active, and the other has less or no activity, such as (−)‐enantiomer of profenofos is 4.3‐ to 8.5‐fold more inhibitory to AChE in vivo than the (+)‐enantiomer (Nillos, Rodriguez‐Fuentes, Gan, & Schlenk, 2007). Former study of sulfoxaflor mostly focused on biological characterization, degradation mechanism (de Albuquerque et al., 2018), and chiral separation of sulfoxaflor in vegetables, fruits and environment samples (Chen et al., 2014a; Chen et al., 2014b, 2016). To our best of knowledge, little is known about the stereoselective dissipation of sulfoxaflor in Puer tea processing.

Therefore, we are aiming to determine the stereoselective dissipation of sulfoxaflor enantiomers in sun‐dry Puer tea, raw Puer tea, and ripen Puer tea processing and to find the main influence of stereoselectivity in different Puer tea processing.

## 2 MATERIALS AND METHODS

### 2.1 Chemical materials

Analytical standard stereoisomer sulfoxaflor (1,000 µg/ml) is provided by J & K scientific Ltd. The enantiomerically pure standards: *2S, 3S*‐sulfoxaflor, *2S, 3R*‐sulfoxaflor are obtained from Shanghai Chiralway Biotech Co., Ltd. The 22% sulfoxaflor suspension concentrate is obtained from Dow AgroScience Co. Ltd.

The stock solutions of the reference compounds are produced by dissolving the compounds in methanol, and the working standard solutions are prepared daily from stock solutions by dilution with the appropriate volume of the mobile phase. All solutions are stored in a refrigerator at −18°C. HPLC‐grade acetonitrile and methanol are provided by Tedia Company Inc. Water is purified using a Milli‐Q system (Millipore). The other solvents, purchased from Shanghai Chemical Factory, are of analytical grade.

Puer tea samples are acquired from the Shunming tea cooperative agency of farmers, Fengshan County, Puer city, China.

#### 2.1.1 Raw Puer tea processing

The raw Puer tea are processed with sun‐dry Puer tea. Twenty kg of sun‐dry Puer tea is sprayed with 1 ml of 22% the formulation product diluted with 1 L water. The raw Puer tea are placed under air temperature (5–28°C). The samples are collected at intervals time on 0 (2 hr), 3, 5, 6, and 8 months. The residues amount expressed as dry sample.

#### 2.1.2 Ripen Puer tea processing

To ferment the ripen Puer tea, 20 kg of sun‐dry Puer teas is sprayed with 5 ml of 22% the formulation product diluted with 5 L water under the conditions of 35% moisture content. During the pile‐fermentation, the fermented tea was artificially turned and piled again at 7 (first pile), 14 (second pile) days, the facial pile‐fermentation tea is splashed by 100 ml water at 3 day intervals. The sample is collected at intervals time on 0 (2 hr), 2, 4, 6, 8, 10, 12, and 14 days. The residues amount expressed as dry sample.

#### 2.1.3 Fermentation temperature of ripen Puer tea processing

To research dissipation during fermentation temperature, the 140 g tea is fermented through 37, 40, and 45°C under incubator in laboratory, and the facial tea is splashed by 20 ml water at 5‐day intervals to keep 35% moisture content during fermentation processing. The sample is collected at intervals time on 1 hr, 3, 6, 9, 12, 15, 18, and 21 days. The residues amount expressed as dry sample.

#### 2.1.4 Sun‐dry Puer tea processing

In normal sun‐dry Puer tea manufacturing, the leaves are harvested through nature wither (half day), roast (1–2 hr), twisting and kneading (half hour), sun‐dry processing. We found the sulfoxaflor degraded faster under sun‐dry period. So to research dissipation during sun, 2 kg of fresh teas are sprayed with 0.5 ml of 22% the formulation product diluted with 1 L water, then spread out, and dried under sun. Samples are collected at intervals time on 0, 2, 4, 6, 8, 10, and 12 hr. Samples are analyzed the moisture content and residues value.

### 2.2 Calculation of degradation kinetics and enantiomer fraction

In the degradation study, the degradation kinetics was calculated according to following equation.

$$y\;=\;C_{o}e^{‐kt}$$

where *C*_o_ and *y* are the concentration of each stereoisomers at time 0 and *t*, respectively. The starting point of the regressive function is the maximum value of the stereoisomer concentration, which decrease in the interval days.

The enantiomer fraction (EF) was calculated as EFx = Exa/(Exa + Exb), Exa referred to concentration of the *2S*, *3S*‐sulfoxaflor or *2R*,*3S*‐sulfoxaflor, Exb referred to concentration of the *2R*, *3R*‐sulfoxaflor or *2S*, *3R*‐sulfoxaflor, *x* = 1 or 2. By analogy, we use diastereomer fraction DF = D1/(D1 + D2) to illuminate the selectivity, D1 referred to concentration of *2S,3S + 2R,3S*‐sulfoxaflor, D2 referred to concentration of four stereoisomer sulfoxaflor.

### 2.3 Sample preparation and analysis

The tea samples are homogenized into powder and are passed through a 40 mesh sieve. The made tea are transported to the laboratory and stored in the refrigerator below −18°C until analysis.

A 1 g portion of the dried sample is weighed in a 50‐ml centrifuge tube, and 10 ml water is sprayed and shaken with hand. Then, 20 ml of acetonitrile, 2 g anhydrous magnesium sulfate and 0.5 g sodium chloride, 1 g primary secondary amine (PSA) are added and mixed. The solution is centrifugated for 5 min at 5,000 *g*. The supernatant is filtered through a 0.22‐µm syringe filter and analyzed by LC‐MS/MS.

### 2.4 LC‐MS‐MS detection

Sample analyses are performed on tandem mass spectrometer AB 4000 (AB Sciex) consisted of a 1290 ultra‐high‐performance liquid chromatograph (Agilent technology). A Chiral Cel OJ‐3R (cellulose tris‐(4‐methylbenzoate) as stationary phase, 150 × 4.6 mm i.d., 3 μm, Daicel Ltd.) is employed for the separation of the analyte and is maintained at 30°C. The mobile phase is comprised of 80% water/20% acetonitrile at a constant flow of 0.3 ml/min. The injection volume is 1 μl. The spectral acquisition is operated in anion electrospray ionization mode, and multiple reactions monitoring (MRM) is utilized: Precursor ion is 275.9 (*m/z*), Product ion is 212.9*/261 (*m/z*), Delustering/Focusing potential is −40/−40 (V), and Collision energy is −21/−17 (V). The temperature and flow rate of drying gas (N2) are 550°C and 8.0 L/min, respectively. The nebulizer pressure is 20 Pa.

### 2.5 Quality control

**FIGURE 1:**
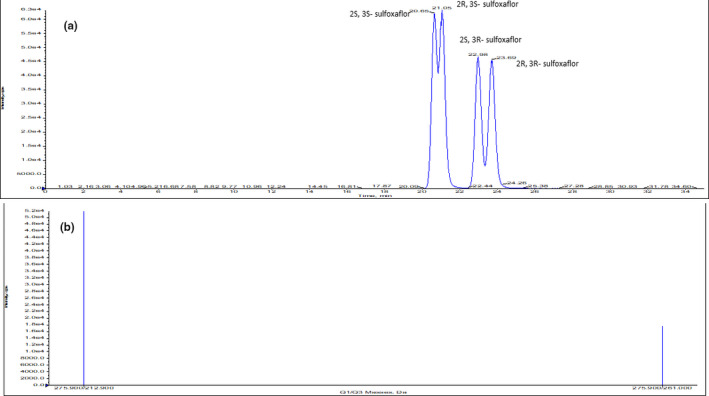
The chromatogram in spiked sample (a) Selective ion chromatogram (b) The MS spectra

Blank sample spiked with working standard solution of sulfoxaflor at three concentration levels (1, 10, 100 µg/kg) is used for method detection limit and recovery assay. Both the blank sample and spiked samples are treated the same as “the sample pretreat and analysis” procedure. The chromatogram of spiked sample is in Figure 1. No sulfoxaflor was detected in the blanks. The mean recoveries are in the range of 80%–92%, 82%–95%, 78%–94%, and 82%–93% for four stereoisomers of sulfoxaflor, respectively. The corresponding relative standard deviation was 10% or below. The linearity was good with the range of 2.0–100 µg/L for each enantiomer, coefficient, *R* ^2^ > .995. The limit of detections (LODs) for the every enantiomers is the concentration that produced three times signal‐to‐noise (*S/N*) ratio, whereas the limit of quantify (LOQ) is based on ten times *S/N* ratio. The LODs for four stereoisomers are 0.5 μg/L, and LOQ is 0.5 μg/kg.

## 3 RESULTS AND DISCUSSION

### 3.1 Stereoselective dissipation of sulfoxaflor under raw Puer tea processing

**FIGURE 2:**
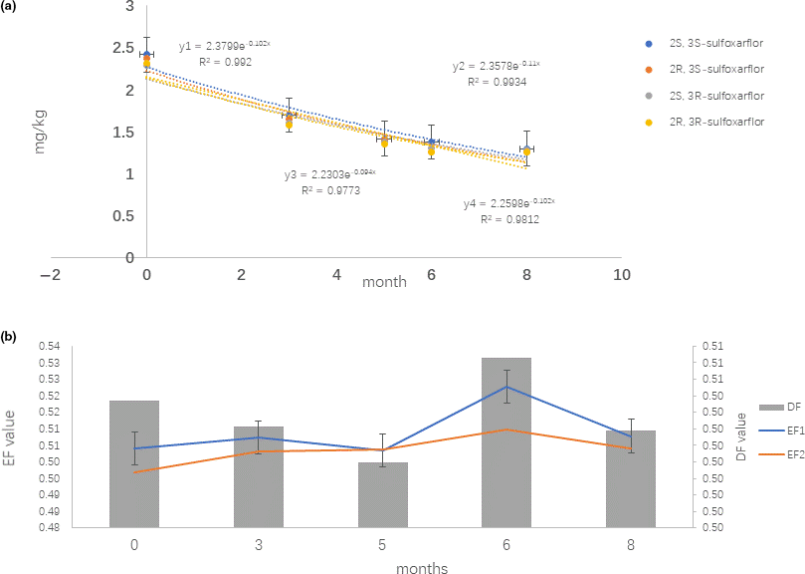
Dissipation of sulfoxaflor stereoisomers (a) and enantioselectivity of EF, DF (b) in raw Puer tea processing

Raw Puer tea is unique due to the microbial fermentation process under nature condition, which may last from several months to many years. To some extent, the quality and flavor of raw Puer tea improves with longer fermentation time, and consequently aged raw Puer tea is more expensive quality (Zhang et al., 2016). The moisture content in raw Puer tea is almost constant with 7%–8%. The concentrations of stereoisomers sulfoxaflor decrease slowly and are <54% of the initial concentrations after 8 months during raw Puer tea processing. The corresponding degradation kinetics are in accord with the pseudo‐first‐order kinetics, and the *R* ^2^ ranged from 0.977 to 0.992 in raw Puer tea processing (Figure 2). The estimated *t*_1/2_ values of the *2S, 3S*‐sulfoxaflor, *2R, 3S*‐sulfoxaflor, *2S, 3R*‐sulfoxaflor, *2R, 3R*‐sulfoxaflor are 6.63, 6.21, 7.04, 6.54 months, respectively. No statistical significance was obtained between the *t*_1/2_ of each enantiomer, with *p* > .05 by Student's paired *t* test at a 95% probability. Stereoselectivity is expressed as EFx. As shown in Figure 2b, the stereoselectivity of EF1 and EF2 is little from 0.50 to 0.52, and the change of DF is small. From the statistical analysis, it showed that no significant enantioselectivity occurred, with P values greater than or close to 0.05 (confidence of 95%). The result is shown that the persistence of sulfoxaflor residue is apparent prolonged under nature temperature and low moisture content. In addition, the degradation of sulfoxaflor is nonenantioselectivity under raw Puer tea processing.

### 3.2 Stereoselective dissipation of sulfoxaflor under ripen Puer tea processing

**FIGURE 3:**
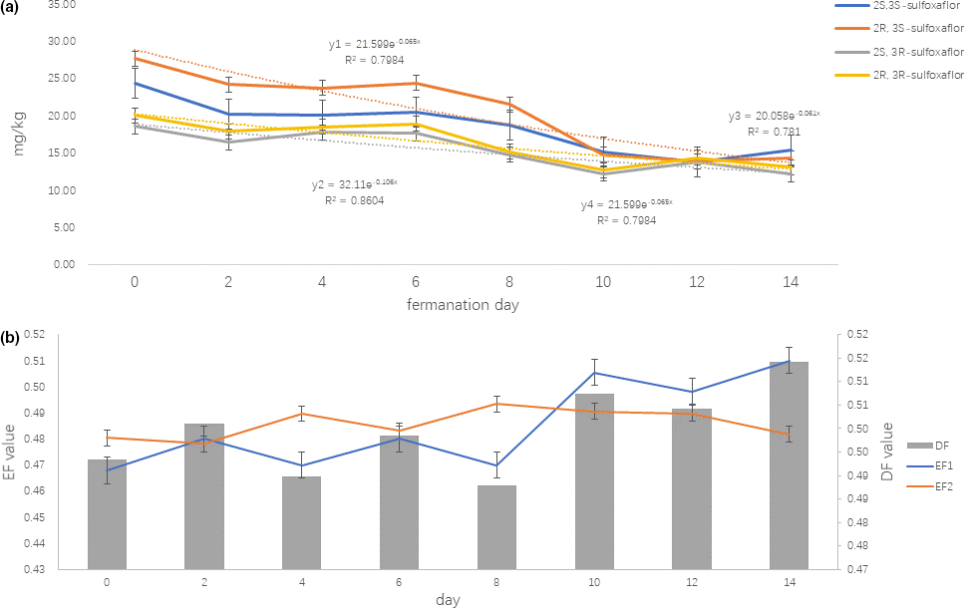
Dissipation of sulfoxaflor stereoisomers (a) and enantioselectivity of EF, DF (b) in ripen Puer tea processing

Tea roasting will play a significant role in reducing the pesticide residue. The piled tea will accelerate the degradation of sulfoxaflor under high temperature and high humidity condition. The corresponding degradation kinetics are in accord with the pseudo‐first‐order kinetics in ripen Puer tea processing (Figure 3), *y*1 = 21.599e^−0.065^ *^X^*, *t*_1/2_ = 8.9 days, *y*2 = 32.11e^−0.106^ *^x^*, *t*_1/2_ = 8.5 days, *y*3 = 20.058e^−0.016^ *^x^*, *t*_1/2_ = 8.2 days, *y*4 = 21.599e^−0.065^ *^x^*, *t*_1/2_ = 8.3 days. The finding shows that for ripen Puer tea, the piled fermentation steps significantly reduce sulfoxaflor content from 6 months (raw Puer tea) to 8 days (ripen Puer tea). EF is a more straightforward representation of stereoselectivity. As shown in Figure 3b, no significant enantioselectivity occurred in ripen Puer tea processing.

### 3.3 Effect of fermentation temperature

Aromatic characteristics in ripen Puer tea are caused by heat and microbial growth at the piled stage (Xu, Yan, & Zhu, 2005). Different fungus steadily increases during the fermentation period with different temperature (Abe et al., 2008).

We investigate three temperatures in the piled stage of ripen Puer tea, of which is controlled below 45°C in piled central temperature. The corresponding degradation kinetics are in accord with the pseudo‐first‐order kinetics in three fermentation temperature (Table 1). Compared with 37°C, the highest persistence of four stereoisomers is observed under 45°C in ripen Puer tea processing. It shows that the degradation of sulfoxaflor residue is quickest under 37°C fermentation temperature. The trend was contrary with the reported paper (Wu, Chu, Wang, & Lur, 2007). It implied that enzyme catalysis may play an more important role in degradation of sulfoxaflor than thermal decomposition. The point will be verified further by more studies.

**TABLE 1:** kinetic parameters for the degradation of stereoisomers at different fermentation temperature

Fermentation temperature/°C	Equation of first‐order kinetics	Correlation coefficient squared (*R* ^2^)	Half‐life *t*_1/2_ (day)
37	*y*1 = 11.568e^−0.143^ *^x^*	.940	4.9
	*y*1 = 13.882e^−0.184^ *^x^*	.922	4.5
	*y*1 = 12.249e^−0.145^ *^x^*	.849	5.7
	*y*1 = 11.768e^−0.13^ *^x^*	.800	5.2
40	*y*1 = 13.967e^−0.037^ *^x^*	.891	22.6
	*y*2 = 15.191e^−0.038^ *^x^*	.902	22.7
	*y*3 = 14.17e^−0.037^ *^x^*	.934	22.3
	*y*4 = 14.694e^−0.039^ *^x^*	.911	21.3
45	*y*1 = 10.745e^−0.018^ *^x^*	.813	37.2
	*y*2 = 11.342e^−0.018^ *^x^*	.761	36.3
	*y*3 = 10.924e^−0.02^ *^x^*	.818	34.3
	*y*4 = 11.031e^−0.018^ *^x^*	.722	35.7

The results of EF and DF were in agreement with those found in raw Puer tea and ripen Puer tea processing, and the degradation of sulfoxaflor was nonenantioselectivity.

### 3.4 Stereoselective dissipation of sulfoxaflor under sun‐dry Puer tea processing

**FIGURE 4:**
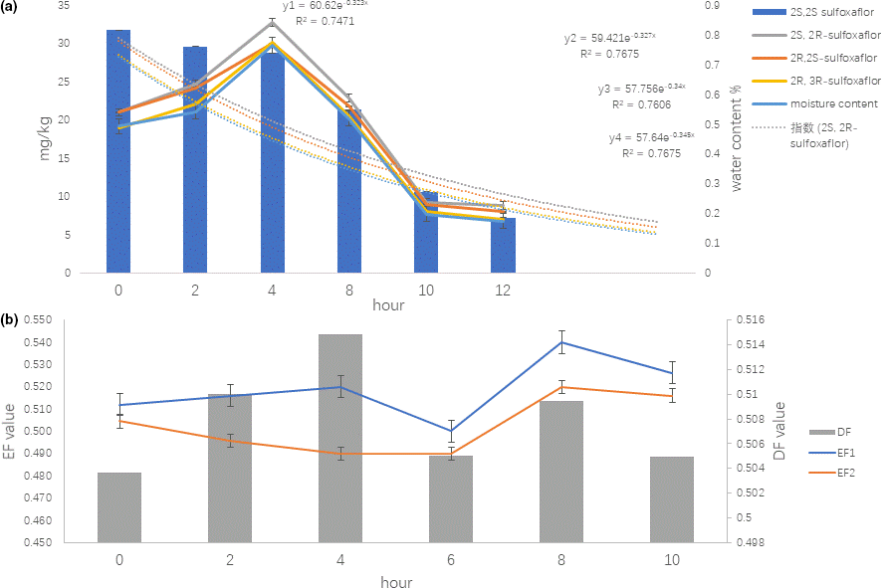
Dissipation of sulfoxaflor stereoisomers (a) and enantioselectivity of EF, DF (b) in sun drying Puer tea processing

Two stages are observed for amount change of sulfoxaflor under sun drying in previous studies (Liu, Jiang, & Li, 2020). Initially, the amount of sulfoxaflor increases to the maximum at 4 hr; then, the concentration decreases gradually and is <58% of the initial concentrations after 10 hr (Figure 4a). Additionally, an interesting phenomenon is observed that the trend is in accordance with moisture content change and the corresponding degradation of sulfoxaflor. The pesticide dissipating under tea processing is mainly from volatility and thermal decomposition to the input of solar radiation (Guillebeau, All, & Javid, 1989). So we infer that the volatility will relate mainly to dissipation of sulfoxaflor. The corresponding degradation kinetics are slightly in agreement with the pseudo‐first‐order kinetics in sun‐dry Puer tea processing, *y*1 = 60.62e^−0.323^ *^x^*, *t*_1/2_ = 4.2 hr, *y*2 = 59.42e^−0.327^ *^x^*, *t*_1/2_ = 4.1 hr, *y*3 = 57.75e^−0.34^ *^x^*, *t*_1/2_ = 4.1 hr, *y*4 = 57.64e^−0.345^ *^x^*, *t*_1/2_ = 4.0 hr, *R* ^2^ is .747–.767. This indicates more faster residue dissipation in sundry tea processing than in greenhouse vegetable with half‐life of 3.5 days (Chen et al., 2016). The EF1 is nearly 0.51 at 2 hr and increased to 0.536 at 8 hr, then decreased to 0.52 at 10 hr (Figure 4b). The results of EF and DF were in agreement with those found in raw Puer tea and ripen Puer tea processing, and the degradation of sulfoxaflor is still nonenantioselectivity in sun‐dry Puer tea processing.

## CONFLICT OF INTEREST

All Authors declare that we have no conflict of interest.

## ETHICAL APPROVAL

This article does not contain any studies with human participants performed by any of the authors.
